# Debriefing after incidents and restrictive practices: a D-FOREST study

**DOI:** 10.1192/j.eurpsy.2025.1526

**Published:** 2025-08-26

**Authors:** B. Walls, A. Lindner, J. Leahy, D. Duffy, P. Mc Laughlin, H. G. Kennedy, M. Davoren

**Affiliations:** 1 Central Mental Hospital, National Forensic Mental Health Service, Portrane, Co. Dublin. K36 FD79, Dublin, Ireland; 2 Johannes Gutenberg-Universität, Mainz, Germany; 3 Psychiatry, Trinity College Dublin, Dublin, Ireland; 4 Skou Professor of Forensic Psychiatry, University of Aarahus, Aarhus, Denmark; 5 Visiting Professor of Forensic Psychiatry, University of Bari ‘Aldo Moro’, Bari, Italy

## Abstract

**Introduction:**

The Council of Europe’s Model Mental Health Act places emphasis on the need for a ‘debriefing’ discussion with patients after incidents of restrictive practices to arrive at a common understanding concerning what happened and to consider future prevention strategies. For the patient, issues may arise that may be subjectively reinforcing or subjectively aversive and staff may feel that the incident has, or does not have, alienating consequences.

**Objectives:**

The aim of this study was to evaluate the consequences for the patient and for staff of incidents and use of subsequent restrictive practices, as a basis for future understanding of what debriefing might usefully include.

**Methods:**

A prospective cohort study was completed whereby incidents were rated using the Dynamic Assessment of Situational Aggression (DASA) and DRILL tool ‘consequences’ scales in the Central Mental Hospital (CMH). The DRILL consequences scale consists of three ladders, ‘re-enforcing’, ‘aversive’ (both rated from the point of view of the patient) and ‘alienating’ (rated from the point of view of ward based staff ). Data were gathered as part of the Dundrum Forensic Redevelopment Evaluation Study (D-FOREST). An omnibus General Estimating Equations model (GEE) was tested with the DRILL ‘consequences’ as dependent prior to dismantling studies.

**Results:**

In this study, the 384 patient-days were in scope, 411 lines of data including 326 patient-days, 85 harmful incidents and 63 incidents of seclusion. In an omnibus GEE with the three-item DRILL consequences scale as dependent variable, DASA on the day before Wald X^2^=3065.9, p<0.001; DRILL behaviours scale Wald X^2^=970.7, p<0.001; DRILL interventions Wald X^2^=140,159.1, p<0.001; DUNDRUM-1 Wald X^2^=1638.9, p<0.001. The three items of the DRILL consequences scale were individually tested as dependent variables in GEE models with DASA the day before, DRILL behaviour scale, DUNDRUM-1 and each of the eight items of the DRILL interventions scale. Only increasing observation levels were not re-enforcing, with searches and seclusion strongly re-enforcing.

**Conclusions:**

We have shown that consequences of harmful behaviours and preventive, restrictive interventions are measurable and proportionate for patients and for staff. Short-term risk on the day prior to an incident was able to predict the re-enforcing and aversive consequences for the patient, but not the alienating consequences – or lack of them – for staff. Future research will examine the way in which the baseline need for security mediates or moderates the relationship between incidents and patients and staff views of the consequences of incidents and restrictive practices.

**Disclosure of Interest:**

None Declared

